# Mechanism and therapeutic potential of targeting cGAS-STING signaling in neurological disorders

**DOI:** 10.1186/s13024-023-00672-x

**Published:** 2023-11-08

**Authors:** Yige Huang, Bangyan Liu, Subhash C. Sinha, Sadaf Amin, Li Gan

**Affiliations:** 1 Helen and Robert Appel Alzheimer Disease Research Institute, Feil Family Brain and Mind Research Institute, Weill Cornell Medicine, New York, NY USA; 2 Weill Cornell Graduate School of Medical Sciences, Weill Cornell Medicine, New York, NY USA

**Keywords:** cGAS, STING, Interferon response, Cytosolic dsDNA, Antiviral, Neuroinflammation, Alzheimer disease, Parkinson disease, ALS

## Abstract

DNA sensing is a pivotal component of the innate immune system that is responsible for detecting mislocalized DNA and triggering downstream inflammatory pathways. Among the DNA sensors, cyclic GMP-AMP synthase (cGAS) is a primary player in detecting cytosolic DNA, including foreign DNA from pathogens and self-DNA released during cellular damage, culminating in a type I interferon (IFN-I) response through stimulator of interferon genes (STING) activation. IFN-I cytokines are essential in mediating neuroinflammation, which is widely observed in CNS injury, neurodegeneration, and aging, suggesting an upstream role for the cGAS DNA sensing pathway. In this review, we summarize the latest developments on the cGAS-STING DNA-driven immune response in various neurological diseases and conditions. Our review covers the current understanding of the molecular mechanisms of cGAS activation and highlights cGAS-STING signaling in various cell types of central and peripheral nervous systems, such as resident brain immune cells, neurons, and glial cells. We then discuss the role of cGAS-STING signaling in different neurodegenerative conditions, including tauopathies, Alzheimer’s disease, Parkinson’s disease, and amyotrophic lateral sclerosis, as well as aging and senescence. Finally, we lay out the current advancements in research and development of cGAS inhibitors and assess the prospects of targeting cGAS and STING as therapeutic strategies for a wide spectrum of neurological diseases.

## Background

DNA serves as a crucial type of damage-associated molecular pattern, capable of activating the innate immune system through interactions with pattern recognition receptors (PRRs). Recognizing DNA is a fundamental mechanism of host defense that involves various PRRs, including Toll-like receptor 9 (TLR9), IFI16, RNA polymerase III, and cyclic GMP-AMP synthase (cGAS) [1–4]. Discovered in 2013, cGAS is a key regulator of innate immune response to double-stranded DNA (dsDNA). Functioning as the primary sensor of cytosolic DNA, cGAS detects both foreign DNA from pathogens and self-DNA released during cellular damage, activating stimulator of interferon genes (STING) and triggering a type I interferon (IFN-I) response. The ability to identify foreign pathogen DNA is essential for combating bacterial and viral infections. However, indiscriminate sensing of self-DNA can lead to sustained activation of the innate immune response, and result in dysregulation of tissue homeostasis.

Originally identified as a crucial component of the innate immune host-defense system, cGAS-STING signaling has more recently been recognized as a key pathway in the initiation and pathogenesis of inflammatory diseases, including cancer, autoimmune and neurodegenerative diseases [5]. The central nervous system (CNS) is a highly regulated and intricate environment and requires tight control of immune responses to maintain neuronal function and repair injury. Recent studies revealed the presence and functional significance of cGAS-STING signaling in various CNS cell types, including resident brain immune cells, neurons, and glial cells. Research interest has been growing in the role of cGAS-STING in the CNS, as neuroinflammation has proved to be a key player in neurological and neurodegenerative diseases [6, 7].

This review aims to provide a comprehensive overview of the current understanding of the cGAS-STING pathway in the CNS in health and disease, with a focus on its involvement in neuroinflammation, neurodegenerative diseases, cellular senescence, and potential therapeutic implications. We hope to provide an update on this emerging field and highlight avenues for future research and therapeutic development for neurological diseases.

## cGAS-STING as a DNA-sensing pathway

**Fig. 1:**
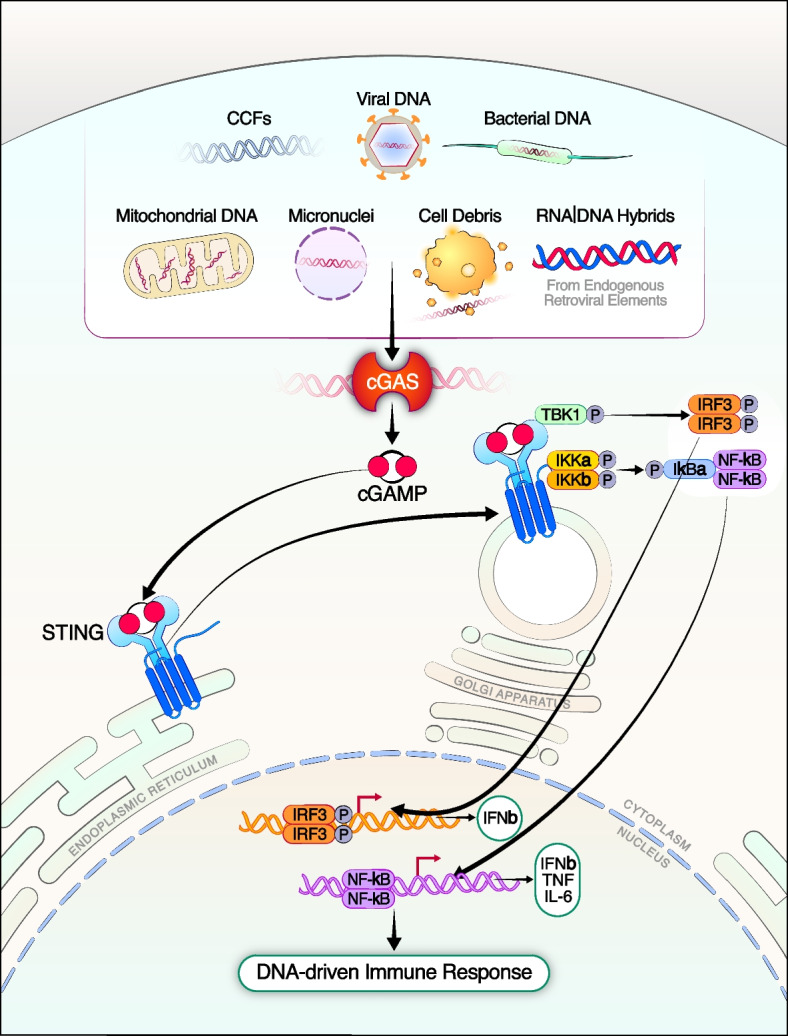
Activation of the cGAS-STING pathway by cytosolic DNA. Foreign (bacterial, viral) or self (mitochondrial, genomic) DNA in the cytosol can be sensed by cGAS, which produces cGAMP from ATP and GTP. cGAMP binds to and phosphorylates STING, inducing STING activation. STING further translocates from the ER to the Golgi apparatus. Phosphorylated STING recruits TBK1 and the IKK kinase, triggering innate immune response, including transcription of IFN-I and other immunomodulatory genes via phosphorylating IRF3 or activating NF-κB, respectively

DNA has well-conserved localizations in the nucleus and mitochondria. Mislocalization of DNA in the cytosol or endosomal compartment is indicative of foreign viral or bacterial infection or nuclear disintegration [5]. The innate immune system contains receptors that detect pathogenic DNA, one of which is cGAS. cGAS was first discovered by Sun et al. for its function as a cytosolic DNA sensor that activates the IFN-I response [8]. cGAS binds to DNA, undergoes switch-like conformational changes at the activation loop, and forms an oligomeric complex with DNA [9] (Fig. 1). Activated cGAS produces cyclic GMP-AMP (cGAMP) from ATP and GTP [4, 10]. Subsequently, cGAMP binds to and activates the STING in the endoplasmic reticulum (ER). STING then translocates to the Golgi apparatus and recruits TANK-binding kinase 1 (TBK1), which phosphorylates STING at Ser366 (in human) or Ser365 (in mice) [11]. Phosphorylated STING, in turn, recruits interferon regulatory factor 3 (IRF3), leading to IRF3 phosphorylation by TBK1 [12]. Upon phosphorylation, IRF3 dimerizes and translocates to the nucleus, ultimately activating expression of IFN-I. In addition to IRF3, STING also recruits and activates the IKK kinase, which phosphorylates IκBα at two N-terminal serines and triggers ubiquitin-dependent IκBα degradation in the proteasome. The degradation of IκBα leads to rapid and transient nuclear translocation of canonical NF-κB, resulting in the production of pro-inflammatory cytokines and chemokines [13] (Fig. 1).

Sources of cytosolic dsDNA that trigger cGAS are diverse. They could originate from microbial pathogens, such as bacteria and DNA viruses, during an infection, as these organisms carry dsDNA in their genomes [14, 15]. In the context of cancer, endogenous DNA, such as the DNA within debris of dead tumor cells, comprises an important component of innate immunity against tumors [16]. Antigen-presenting cells (APCs) can engulf tumor cells, and tumor-cell-derived DNA activates APCs through the cGAS-STING pathway. Moreover, self-DNA from the host cell also accumulates in the cytoplasm and activates cGAS, under conditions of cellular stress or damage when nuclear or mitochondrial DNA is mislocalized to the cytosol. For instance, the loss of genomic integrity from DNA replication or damage can lead to formation of micronuclei. The rupture of these micronuclei releases chromosomal DNA into the cytosol, triggering cGAS activation [17]. Similarly, the accumulation of cytoplasmic chromatin fragments (CCFs) can occur through autophagic removal of nuclear DNA or DNA leakage from the nucleus during cellular senescence and aging, leading to cGAS activation [18]. In addition to nuclear DNA, mitochondrial stress or damage can result in the release of mitochondrial DNA (mtDNA) into the cytosol, further activating the cGAS-STING pathway [15]. Notably, cGAS is not limited to sensing dsDNA; it can also detect RNA:DNA hybrids, which can originate from retrotranscription of retroviruses or endogenous retroviral elements, among other potential sources [19, 20].

### Mitochondrial DNA

Mitochondria are unique organelles that contain their own genome that encodes genes essential for normal mitochondrial function. When released into cytosol, mitochondrial DNA (mtDNA) serves as a type of damage-associated molecular pattern (DAMP) that can be recognized by the innate immune system. mtDNA release into the cytosol can be induced by various triggers of mitochondrial damage, such as oxidative stress, mitochondrial dysfunction, and viral infection.

Oxidative stress within mitochondria occurs due to the excessive production of reactive oxygen species (ROS), primarily generated in the mitochondrial matrix, where mtDNA resides, during the process of oxidative phosphorylation. Mitochondrial oxidative stress leads to release of oxidized mtDNA to the cytosol, triggering innate immune signaling, such as IFN-I and NLRP3 inflammasome activation [21–23]. Transcription factor A, mitochondrial (TFAM), is an abundant mtDNA-binding protein that regulates the nucleoid structure, abundance, and segregation of mtDNA. West et al. [15] discovered that mtDNA stress caused by TFAM deficiency and herpes simplex virus (HSV-1) infection can cause mtDNA to be released to the cytosol. Cytosolic mtDNA then engages the DNA sensor cGAS, promotes STING–IRF3-IFN-I signaling, and induces interferon-stimulated gene (ISG) expression [15]. Mitophagy, a vital process responsible for the removal of damaged mitochondria through autophagy, plays a crucial role in preventing mtDNA leakage and subsequent innate immune activation. Parkin, an E3 ubiquitin ligase, and PINK1, a ubiquitin kinase, are involved in mitophagy function and are risk genes for early onset Parkinson’s disease (PD). Parkin or PINK1 deficiency impairs mitophagy, inducing mtDNA release, and activation of the cGAS-STING IFN-I response [24].

The precise mechanisms underlying the release of mtDNA from mitochondria to the cytosol are not fully understood, but several mechanisms have been reported. During apoptosis, the BCL-2-associated X protein (BAX)- and BCL-2 homologous antagonist/killer (BAK) become activated and induce mitochondrial outer-membrane permeabilization. This leads to release of mtDNA that is recognized by the cGAS-STING pathway [25, 26]. mtDNA fragments can also be released from the mitochondrial permeability transition (MPT) pore [27, 28]. In amyotrophic lateral sclerosis (ALS)**,** TDP-43 accumulates in the mitochondria of neurons and causes mtDNA release into the cytoplasm via the MPT pore, leading to cGAS-STING activation and the upregulation of NFκB and IFN-I pathways [29]. Additionally, during oxidative stress, mtDNA can escape through macropores formed by oligomerization of voltage-dependent anion channels (VDACs) and trigger IFN-I response [30].

In summary, the leakage of mtDNA serves as a common source of cytosolic DNA that can be sensed by cGAS, linking mitochondrial function with innate immunity.

### Genomic DNA

Genomic DNA is strictly compartmentalized within the nucleus to prevent autoimmunity. However, in cases of DNA damage and genomic instability, DNA can be exposed to the cytosol by forming micronuclei. Micronuclei occur due to mis-segregation of DNA during cell division and consist of chromatin surrounded by the nuclear envelope. in a mouse model characterized by nuclease RNase H2 deficiency, cGAS localizes to micronuclei arising from genomic instability. Additionally, cGAS colocalizes with γH2AX, a marker of DNA damage. Micronuclei formation due to radiation-induced DNA damage activates cGAS in a cell-cycle-dependent manner and induces ISG upregulation and proinflammatory response [17].

CCFs are another type of cytoplasmic chromatin commonly observed in senescent cells. During senescence, the expression of cytoplasmic DNases, such as DNase2 and TREX1, is downregulated, resulting in the cytoplasmic accumulation of nuclear DNA and aberrant activation of the cGAS-STING pathway [31].

Further, RNA:DNA hybrids, such as R-loops, form during transcription and are restricted to the nucleus under normal physiological conditions [32]. However, dysregulation of R-loop processing, such as loss of function mutation in RNA–DNA helicase senataxin (SETX), leads to the accumulation of cytoplasmic RNA:DNA hybrids [33]. These hybrids bind to the pattern recognition receptors cGAS and TLR3, activating IRF3 and inducing apoptosis. Cytoplasmic hybrids have been observed in fibroblasts derived from patients of ataxia oculomotor apraxia type 2, a type of neurodegenerative disease caused by mutations in SETX. These hybrids have been shown to activate IFN-I response in a cGAS/TLR3-dependent manner. R-loops also participate in DNA repair at dsDNA breaks [34]. Dysregulation of R-loop formation can lead to DNA damage and genomic instability, further triggering innate immune signaling.

Transposable elements, also known as “jumping genes”, are DNA fragments that move within/between genomes. Retrotransposons, a type of transposable element, duplicate through RNA intermediates by reverse transcription and are inserted at new genomic locations. Long-interspersed element-1 (LINE-1) is the only human retrotransposon capable of autonomous retrotransposition. During aging and cellular senescence, LINE-1 becomes transcriptionally derepressed, resulting in the accumulation of cytoplasmic RNA:DNA hybrids due to retrotranscription of LINE-1 elements and activating IFN-I response through cGAS-STING [19, 35].

### Viral DNA

Viral infection is a common source of cytosolic DNA recognized by the innate immune system. For instance, Infection by HSV-1, a DNA virus, can lead to acute viral encephalitis. HSV potently induces IFN-I response in the CNS, with microglia being the major cell type [14]. The induction of IFN-I depends on the cGAS-STING pathway as cGAS- and STING-deficient mice are more susceptible to HSV-1 encephalitis after peripheral infection. This susceptibility is correlated with impaired IFN-I expression in the CNS. Similarly, STING deficiency increases HSV-1 replication in microglia in vivo. Besides DNA viruses, retroviruses, such as HIV, are a type of virus capable of inserting a DNA copy of its RNA genome into the host genome. Reverse transcribed HIV DNA has been reported to trigger the innate immune response through the cGAS-STING and IFN-I pathway [36].

## cGAS-STING in different cell types in the central and peripheral nervous systems

cGAS and STING are widely expressed in both immune and non-immune tissues and cell types. Most studies on cGAS-STING in the brain focus on microglia, the brain’s resident immune cell type with the highest expression of the cGAS-STING proteins and the most direct functional role. However, cGAS-STING is also important in other non-immune cell types.

### Microglia

Neuroinflammation is the activation of an inflammatory response in the brain and is characterized by the expression of ISGs, production of proinflammatory cytokines, chemokines, ROS, and secondary messengers [37]. Microglia play a prominent role in neuroinflammation. When activated, microglia release inflammatory cytokines such as IL-1β, IL-6, and TNF-α, as well as chemokines like CCL2, CCL5, and CXCL10. These signaling molecules can be sensed by other cell types in the brain, including astrocytes and neurons, thereby propagating the inflammatory response [38]. Sustained pathological neuroinflammation can lead to various detrimental effects, including peripheral immune cell infiltration, increased permeability and breakdown of the blood–brain barrier (BBB), demyelination, synaptic loss, and neuronal death. Neuroinflammation is commonly observed in conditions involving CNS injury, neurodegeneration, and aging.

IFN-I cytokines are essential in mediating neuroinflammation. They act through the IFN-I receptor (IFNAR), which is commonly expressed by microglia, astrocytes, endothelial cells, and neurons. The binding of IFN-I to IFNAR activates the Janus kinase (JAK)/signal transducer and activator of transcription (STAT) signaling cascade to elicit an anti-viral, anti-proliferative and immunostimulatory response through ISG induction. This cascade results in the secretion of proinflammatory cytokines and chemokines and more IFNs [39]. The cGAS-STING pathway directly activates IFN-I by sensing cytosolic DNA. In the brain, cGAS and STING are predominantly expressed in microglia as innate immune regulators. The cGAS-STING pathway in microglia is responsible for IFN-I induction in HSV-1 infection [14]. More importantly, this pathway has also been implicated in various conditions characterized by maladaptive neuroinflammation, including neuroinflammatory diseases, neurodegenerative disorders, and the aging process.

### Endothelial cells

cGAS-STING signaling has been observed to be activated in endothelial cells of human patient skin and lung tissues following infection with SARS-CoV-2, the RNA virus responsible for the global COVID-19 pandemic [40]. This activation occurs in part due to defective mitochondria in the infected endothelial cells, which release mtDNA into the cytosol. cGAS recognizes mtDNA, leading to IFN-I production and endothelial cell death. Clinical studies have confirmed cognitive deficits in patients who have recovered from COVID-19, pointing to potential neurological consequences [41]. To understand the potential driver of cognitive decline, further studies in the brain revealed elevated levels of IFN-I and IFN-γ responses in the choroid plexus epithelium after SARS-Cov-2 infection. This response leads to the release of IFN-β, complement proteins, and CCL/CXCL chemokines, subsequently activating glial cells [42, 43]. The activation of endothelial cGAS and IFN-I signaling appears to be closely linked to COVID-19-related cognitive deficits. Thus, it is important to determine whether brain endothelial cGAS-IFN-I contributes to various pathological aspects such as BBB breakdown, peripheral immune cell infiltration, and chronic inflammation in different CNS diseases.

### Nociceptors

Nociception is the detection of noxious stimuli by peripheral sensory neurons known as nociceptors in the dorsal root ganglia. These nociceptors are recognized as critical regulators of inflammation and immunity. Nociceptors express a large array of damage- or pathogen-associated molecular pattern-sensing PRRs, one of which is STING. STING regulates steady-state nociception by preventing nociceptor hyperexcitability through IFN-I signaling. Mice lacking STING or IFN-I signaling exhibit hypersensitivity to nociceptive stimuli and heightened nociceptor excitability. In contrast, the intrathecal administration of STING agonists leads to an IFN-I response and suppresses pain and nociceptor excitability in mice and non-human primates [44].

### Dorsal root ganglion neurons

In the peripheral nervous system, the dorsal root ganglion neurons spontaneously regenerate their peripheral axons after nerve injury, and the innate immune response is critical for axon regeneration. IFNγ secreted by injured axons upregulates cGAS expression in Schwann cells and blood cells. cGAS produces cGAMP in response to DNA damage that acts an immunotransmitter and activates neuronal STING to promote axon regeneration [45].

### Other CNS cell types

cGAS and STING may be expressed in CNS cell types other than microglia, such as neurons and astrocytes [14, 46, 47]. However, studies have shown that microglia may employ a STING-dependent antiviral mechanism that differs from that of astrocytes and neurons. Reinert et al. [14] reported that, in response to HSV-1 infection, genetic deletion of STING significantly increased the viral replication in microglia, but not astrocytes or neurons, suggesting that microglia possess a distinct STING-dependent antiviral response. Microglia also exhibit a more potent IFN-I response to HSV infection compared to astrocytes, while neurons fail to induce any IFN-I response. This variation in the ability to induce the IFN-I response correlates with the levels of cGAS and STING expression. Furthermore, in response to HSV-1 infection, microglia can transmit STING-dependent antiviral signals to neurons and prime the Toll-like receptor 3 (TLR3) pathways in astrocytes through paracrine signaling [14].

In a recent study performed in a model of tauopathy characterized by the accumulation of tau aggregates induced by the expression of a mutant tau carrying frontotemporal dementia (FTD)-linked mutation P301S [47], single-nuclei RNA sequencing of hippocampi suggested that *Cgas* and *Sting* (*Tmem137*) are predominantly expressed in microglia. Although they can also be detected in other cell types, such as endothelial cells, the IFN-I response induced by tau was mostly observed in microglia [47]. In line with this observation, Xie et al. [46] demonstrated that oligomeric human Aβ42 peptide treatment led to increased levels of circulating mtDNA and 2′3′-cGAMP (direct product of cGAS activation) in primary cell cultures of microglia, neurons and astrocytes. However, an increase of IFN-β level was only found in the supernatants of primary microglia but not in primary neurons or astrocytes [46]. These results suggest that cGAS activation can be induced in multiple neural cells by Aβ oligomers, but microglia elicit a more specific STING-IFN response. They further suggest that the STING-IFN-I response is predominantly observed in microglia, which could then transfer inflammatory signaling to other neural cells [46]. However, more research using conditional knockout models is needed to further explore whether cGAS and STING play functional roles in neurons and astrocytes.

These studies collectively shed light on the role of the cGAS-STING pathway in various cell types, particularly non-immune cells, such as endothelial cells and peripheral neurons. The cell-type-specific roles of cGAS-STING in maintaining CNS homeostasis and contributing to disease development require further investigation. In the following sections of the review, we will focus on the involvement of cGAS-STING in diseases of the CNS.

## cGAS-STING contributes to CNS diseases and aging

**Fig. 2:**
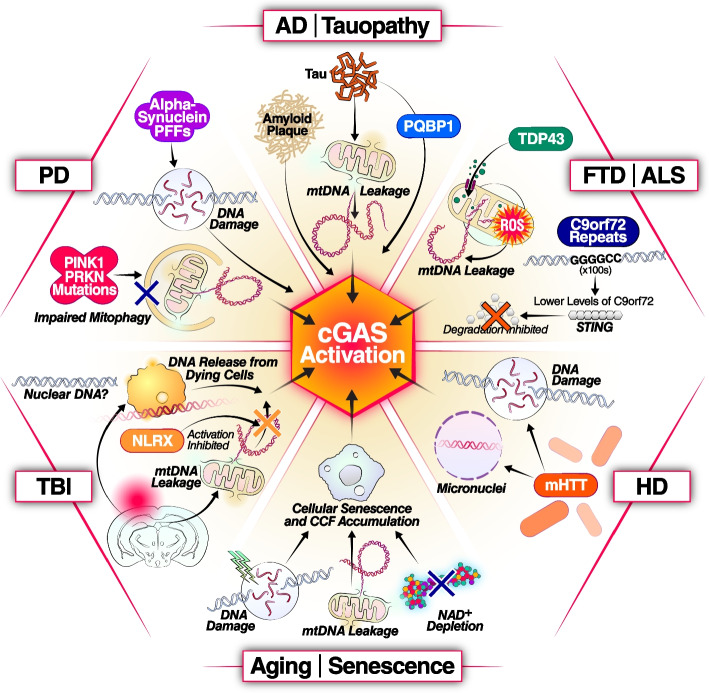
cGAS activation in different CNS diseases and conditions. Activation of cGAS-STING-IFN-I signaling is a convergent pathway in various CNS diseases and conditions. In AD, the accumulation of tau tangles and amyloid plaques can lead to the activation of cGAS [46–48]. In PD, PINK1 and PRKN mutations and αSyn preformed fibril (αSyn-PFF) can induce cGAS activation [24, 49]. In ALS, Mutant TDP-43 protein can activate cGAS [29]. C9orf72 mutation leads to impaired degradation of STING, causing activation of the IFN-I response [50]. In Huntington’s disease (HD), cGAS activation is induced by DNA damage caused by mutant HTT protein (mHTT) [51]. In traumatic brain injury (TBI), traumatic impact induces DNA damage and neuronal death, activating cGAS by DNA released from dying neurons [52, 53]. In the context of aging and senescence, the activation of cGAS-STING is an important contributor to the development of senescence and senescence-associated secretory phenotype (SASP) [18, 54–57]

Mislocalized DNA has been suggested as a converging driver across CNS disease pathogenesis by inducing neuroinflammation through the cGAS-STING pathway. Here we review the disease-specific role of cGAS-STING activation in the CNS (Fig. 2).

### Parkinson’s disease

PD is a neurodegenerative movement disorder characterized by neuronal loss in the substantia nigra, which results in striatal dopamine deficiency and intracellular inclusions of α-synuclein (αSyn) aggregates. The activation of IFN-I signaling was observed in postmortem PD human samples and in the 1-methyl-4-phenyl-1, 2, 3, 6-tetrahydropyridine (MPTP) mouse model. Deletion of the IFN-I receptor (*Ifnar1*^*−/−*^) reduced MPTP-induced IFN-I response, activated microglia (CD11b, CD86, CD32, CD16), and rescued the loss of tyrosine hydroxylase (TH)-positive dopaminergic neurons, suggesting the deleterious role of IFN-I signaling in PD pathogenesis [58].

Activation of cGAS-STING signaling is one of the sources of IFN-I response. Mutations in PARKIN and PINK1 are associated with early-onset PD. Sliter et al. [24] reported that exhaustive exercise induces acute mitochondrial stress and triggers the release of mtDNA in mice with deficient mitophagy caused by Parkin (*Prkn* ^*−/−*^) or PINK1 (*Pink1* ^*−/−*^) deletion. This leads to activation of cGAS-STING and inflammation, including an increase in inflammatory cytokines in the blood serum [24]. In a separate model of Parkin deficiency with chronic mtDNA mutation (*Prkn* ^*−/−*^;*mutator),* the loss of STING prevented inflammation, motor defects, and dopaminergic neuron degeneration [24]. Thus, impaired mitophagy, which causes mtDNA release into the cytosol, serves as a major activator of cGAS-STING in these PD models.

In an αSyn-preformed fibril (αSyn-PFF)-treated primary cell–culture model of PD, αSyn-PFFs induced double-stranded DNA break (DSB) damage response, characterized by γH2A.X and TBK1 activation, in primary microglia-astrocyte cultures. Similarly, in the αSyn-PFF mouse model, TBK1 activation and DNA damage within striatal microglia were observed prior to the onset of dopaminergic neurodegeneration. STING inactivation in the αSyn-PFF mice attenuated IFN-I signaling, α-Syn PFF-induced motor deficits, pathological αSyn accumulation, and dopaminergic neuron loss [59].

### Amyotrophic lateral sclerosis

ALS is characterized by the degeneration of motor neurons, which leads to muscle weakness and eventual paralysis. Cytoplasmic accumulation of TAR DNA-binding protein of 43 kDa (TDP-43) is a hallmark of ALS. Yu et al. [29] detected cGAS-STING activation in motor neurons derived from induced pluripotent stem cells (iPSCs) from patients with mutations in *TARDBP* (encoding TDP-43) and a mouse model of ALS with human mutant TDP-43 overexpression (Prp-TDP-43^A315T^). Mutant TDP-43 invades mitochondria, leading to mitochondrial destabilization and release of mtDNA into the cytoplasm via the mPT pore. STING deletion in the TDP-43 mutant mice increased their lifespan by 40% without affecting TDP-43 expression, decreased neuroinflammation (e.g., IFN-I and NFκB gene expression in the cortex and spinal cord) and rescued the loss of layer V cortical neurons. Similarly, pharmacological inhibition of the STING in vivo using STING inhibitor H-151 significantly reduced IFN-I and NFκB gene expression and prevented the loss of cortical neurons [29].

Expansions of a hexanucleotide repeat (GGGGCC) in the *C9orf72* gene are the most common cause of familial ALS and frontotemporal dementia (FTD) (C9-ALS/FTD). McCauley et al. [50] reported that dendritic cells from *C9orf72*^-/-^ mice show early activation of the IFN-I response through STING. Degradation of STING through the autolysosomal pathway is diminished in *C9orf72*^−/−^ myeloid cells. Blocking STING suppresses hyperactive IFN-I responses in *C9orf72*^-/-^ immune cells. Furthermore, an elevated IFN-I signature was observed in blood-derived macrophages and brain tissue of patients with C9-ALS/FTD. Treatment of patient peripheral blood mononuclear cells with a STING inhibitor suppressed the elevated IFN-I response [50].

Mutations in superoxide dismutase 1 (SOD1) are found in some inherited forms of ALS. The activation of IFN-I response was reported in the human SOD1(G93A) transgenic mouse model of ALS. Specifically, an upregulation of ISGs was observed in astrocytes surrounding motor neurons in the spinal cord [60]. Exploring the therapeutic potential of suppressing cGAS-STING in ALS is an exciting avenue for future research and may offer new insights into ALS treatment strategies.

### Huntington’s disease

HD is caused by a dominantly inherited CAG (cytosine-adenine-guanine) repeat expansion mutation of the huntingtin gene (*HTT*). The mutant HTT protein (mHTT) is ubiquitously present and affects multiple cellular processes, including transcriptional regulation, microtubule-based transport, and proteostasis, leading to damage in the brain’s striatum and resulting in motor, psychiatric, and cognitive deficits [61]. Inflammation has been implicated in the pathogenesis of HD, as proinflammatory cytokines (such as IL6, IL-8, and TNF-α) are elevated in the plasma of HD patients and correlate with disease progression [62]. Furthermore, microglial activation and an increased number of microglia were reported in HD clinical studies and postmortem brain samples [63, 64].

Genome-wide association studies have determined that pathways involved in DNA handling and repair are associated with somatic mutations due to genomic instability and disease progression in HD [65]. By linking DNA damage and inflammatory responses in HD, Sharma et al. [51] reported that cGAS mRNA is selectively upregulated in mouse HD striatal cells, with high levels of ribosome occupancy at exon 1, resulting in increased cGAS protein expression. cGAS activation is induced by micronuclei in the cytoplasm of both mouse HD striatal cells and human embryonic stem cell–derived HD neurons. The activation increases both inflammatory and autophagy responses through autophagy initiators LC3A and LC3B. Further, cGAS activity is increased in HD striatum as indicated by increased phosphorylation of STING and TBK1. These cause the upregulation of inflammatory genes, including *Ccl5* and *Cxcl10*. In contrast, cGAS depletion using CRISPR/Cas9 inhibited inflammatory gene expression and autophagy flux in HD striatal cells [51].

### Alzheimer’s disease

Alzheimer’s disease (AD) is the most common form of dementia and is characterized by amyloid-β (Aβ)-containing extracellular plaques and tau-containing intracellular neurofibrillary tangles. Sustained neuroinflammation in response to pathogenic proteins through activation of immune cells, especially microglia, has been recognized as another hallmark of AD [38]. Aligned with the proinflammatory signature in AD, IFN-I response and cGAS signaling are involved in multiple models of AD.

Roy et al. [66] discovered the prevalent activation of IFN-I response across multiple mouse models of brain amyloidosis. Importantly, in the 5xFAD model, microglia became attracted to nucleic acid–containing Aβ plaques and switched to a Clec7a^+^ neurodegenerative phenotype. The blockade of IFNAR with an IFNAR antibody, on the other hand, significantly dampened the activation of microglia, suggesting the involvement of nucleic-acid sensing innate immune pathway in the pathogenesis of AD [66]. Consistent with this report, Xie et al. [46] reported that cGAS binds cytosolic dsDNA and becomes activated in human AD brains. In 5xFAD mice, immunostaining showed colocalization of phosphorylated STING and IRF3 with activated microglia marker CD68 around Aβ plaques in the dentate gyrus, suggesting cGAS-STING activation in the microglia. cGAS deletion alleviated cognitive impairment and reduced Aβ pathology and ISG expression in the hippocampus. Further, cGAS deletion inhibited the formation of neurotoxic reactive astrocytes. Aβ-induced neurotoxicity was partially rescued upon treatment with *Cgas*^*−/−*^ astrocyte-conditioned media. Thus, cGAS-STING may regulate the neuroinflammatory response through a microglia-astrocyte-neuron tri-cellular signaling pathway [46].

Intracellular misfolded tau tangles are another hallmark of AD. Tau proteins were found to interact with polyglutamine binding protein 1 (PQBP1), a sensor of the cGAS-dependent innate response to HIV-1 by binding to reverse-transcribed HIV-1 DNA and interacting with cGAS. Extrinsic tau proteins are recognized by PQBP1 in microglia, activating cGAS-STING and inducing nuclear translocation of NFκB in vitro. In vivo microglia-specific deletion of *Pqbp1* abolished the colocalization of PQBP1 to tau, nuclear translocation of NFκB or cGAS recruitment, reduced expression of inflammatory stimulated genes, and rescued a cognitive deficit in an acute tau protein injection model [48].

**Fig. 3:**
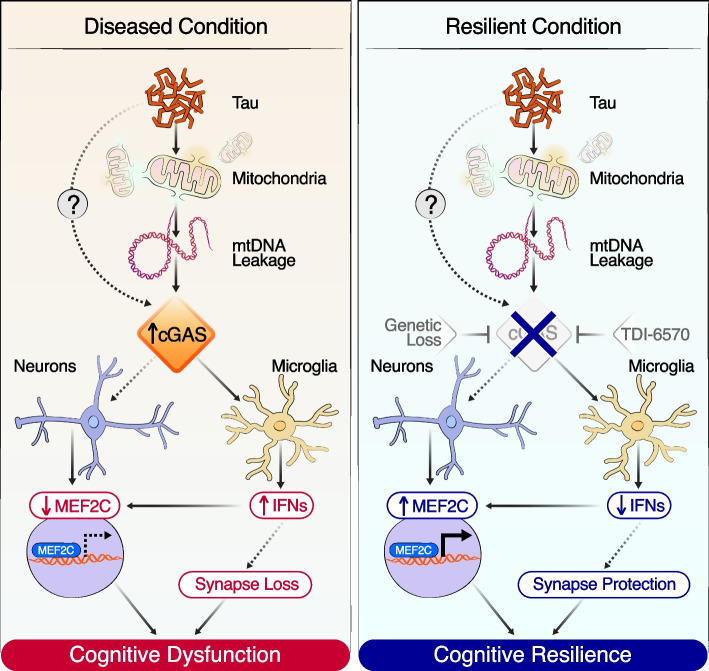
Targeting cGAS preserves MEF2C-mediated cognitive resilience against tauopathy. In disease or vulnerable conditions, pathogenic tau activates the cGAS-dependent IFN response via mtDNA leakage in microglia and a reduction of the MEF2C transcriptional network in excitatory and inhibitory neurons, resulting in cognitive dysfunction. Loss of cGAS through genetic deletion or pharmacological inhibition reduces the IFN response in microglia and enhances the MEF2C transcriptional network in neurons, resulting in cognitive resilience

Pathological hallmarks of AD can precede cognitive decline for decades, yet the molecular mechanisms that confer resilience to neurons during this intermediate period are not well understood. In a recent study [47], we found that cGAS-STING is activated in the P301S tauopathy mouse model and human AD brains harboring high levels of tau pathology. Pathogenic tau activates cGAS in microglia, in part by triggering the leakage of mtDNA into the cytosol (Fig. 3). Microglial cGAS-STING activation leads to the secretion of IFN-inducible cytokines, such as CXCL10 and CCL5, and upregulation of ISGs, such as *Stat1*, *Trim30a*, and *Ddx60*. Genetic deletion of *Cgas* in tauopathy mice mitigated tauopathy-induced microglial IFN-I and protected against synapse loss, synaptic plasticity, and cognitive deficits. *Cgas* deletion did not, however, affect the level of tau pathology in the brains of P301S mice. SnRNA-seq analysis of excitatory and inhibitory neuronal populations in P301S *Cgas*^*−/−*^ hippocampi revealed a robust restoration of transcriptional targets of MEF2C, a key transcription factor in conferring cognitive resilience against AD pathology. Our analysis revealed that microglial IFN-I negatively impacted neuronal expression of MEF2C and its target genes in tauopathy. We further characterized a brain-permeable cGAS inhibitor and showed that pharmacological inhibition of cGAS protected against cognitive impairment in P301S tauopathy mice and enhanced neuronal MEF2C transcriptional network [47].

To explore the therapeutic potential of our findings, we synthesized a human cGAS inhibitor and applied it to human iPSC-differentiated microglia-like cells treated with tau fibrils. Inhibition of cGAS blocked STING phosphorylation and the IFN-inducible cytokines CXCL10 and CCL5 in response to tau, supporting cGAS inhibition as a promising therapeutic approach to improve cognition in AD [47]. Our study uncovered a crucial link between neuronal resilience network and innate immune signaling. While MEF2C was identified as a key factor in conferring cognitive resilience, the neuronal C–C chemokine receptor type 5 (CCR5) signaling was found to have a negative impact on resilience, and its activity tends to increase with aging, leading to impaired memory function [67]. CCL5, the ligand for CCR5, is a chemokine robustly induced by cGAS activation and IFN-I. The activated microglial CCL5 to neuronal CCR5 signaling exacerbates protein aggregation in tauopathy [68]. Understanding neuroimmune interactions will open novel opportunities for unraveling the complexities of and developing new treatments for CNS diseases.

### Traumatic brain injury

TBI arises from a primary external impact to the brain and a delayed, secondary injury consisting of a cascade of molecular, chemical, and inflammatory responses and leading to brain damage and neurological deficits [69]. In a model of TBI induced by closed cortical impact, DNA damage and neuronal death were observed in the cortical tissue 24 h post-injury. cGAS and STING protein and *Ifnb* mRNA levels were increased in the cortex at 72 h post-injury. STAT1 and interferon regulatory factors, including IRF1, IRF3, IRF4, IRF5, and IRF7, were upregulated in both cortex and hippocampus post-injury. In contrast, IFN-β-deficient (*Ifnb*^*−/−*^) mice exhibited a reduced IFN-I response and proinflammatory response after TBI. Moreover, *Ifnb*^*−/−*^ TBI mice showed lower levels of long-term microglial activation, motor, and cognitive deficits than wildtype mice [70]. A follow-up study investigated the effect of aging on inflammatory responses post-TBI, revealing age-related cGAS activation. Specifically, older mice exhibited a robust increase in cGAS protein expression and an enhanced IFN-I signature in the cortex after TBI compared to young TBI mice [52].

Another recent study confirmed that after controlled cortical impact (CCI) injury, there was an increased expression of STING and ISGs [53]. Importantly, the results showed that *Cgas*^*–/–*^ and *Sting*^*–/–*^ mice exhibited reduced neural damage and had a subdued inflammatory response 24 h after the injury [53]. Moreover, *Cgas*^*–/–*^ mice exhibited decreased motor issues four days post-injury, and diminished tissue damage was observed in both groups up to 14 days post-injury. In contrast, the deletion of NLR containing X1 (NLRX1), a negative regulator of STING-TBK1 activation, exacerbated the expression of ISGs and injury in CCI [53]. Interestingly, the evidence supports that elevated mtDNA in the cytosol serves as the major trigger of cGAS-STING in TBI, consistent with findings in tauopathy and TDP43-associated ALS/FTD models.

In summary, these studies indicate that the activation of the cGAS-STING pathway is a converging mechanism contributing to various CNS diseases by mediating chronic neuroinflammation. Thus, targeting the cGAS-STING pathway holds significant therapeutic potential for a wide spectrum of CNS diseases that currently lack effective treatments.

### Senescence and aging

Cellular senescence is a natural process, whereby cells enter an irreversible state of cell-cycle arrest, limiting the growth of abnormal cells and serving as a critical mechanism to prevent tumorigenesis [71]. Senescent cells exhibit several hallmarks, including the upregulation of senescence-associated β-galactosidase (SA-β-Gal) activity and the induction of a senescence-associated secretory phenotype (SASP) [72]. The SASP comprises an array of signaling factors, including NFκB-dependent pro-inflammatory cytokines, such as interleukin 6 and 8 (IL6 and IL8). These cytokines serve a dual role. On one hand, they signal the immune system to remove senescent cells, a crucial step in maintaining tissue health. On the other hand, these cytokines can propagate senescence in neighboring cells, potentially leading to the spread of senescence throughout tissues. Another characteristic of senescent cells is the resistance to programmed cell death through senescent cell anti-apoptotic pathways (SCAPs) [73, 74], which necessitates active clearance of senescent cells by the immune system. Nevertheless, senescent cells persist and accumulate within the numerous tissues, as the capacity of the immune system diminishes with age [75, 76].

Studies have explored the potential of senolytic drugs that target SCAPs to eliminate senescent cells. Animal models have shown that the removal of senescent cells through senolytic therapy can alleviate symptoms associated with aging-related diseases, including AD [77–79]. Notably, a phase I clinical trial evaluated the feasibility of senolytic therapy in mild AD, and the results suggest safety and tolerability of the therapy in AD patients [80]. These studies suggest a potential connection between senescence and the onset of age-dependent neurodegenerative disorders [81].

Senescence can be included by a myriad of environmental triggers (e.g., telomere attrition, mitochondrial dysfunction, and chronic inflammation). Although the mechanisms by which variable stressors induce senescence are distinct, the presence of cytosolic DNA, in the form of CCFs or micronuclei, is a key factor in the development of senescence and SASP [82, 83]. The cGAS-STING pathway is a pivotal component in this process, acting as a central convergence point for senescence triggered by diverse stressors and pathways. The deletion of cGAS reduced the senescence signatures, including SA-β-Gal signaling and SASP, induced by DNA damage in mouse fibroblasts [54]. Meanwhile, deletions of cGAS and STING also diminish senescent phenotypes induced by oxidative stress and cell-cycle arrest [18]. Furthermore, the cGAS-STING pathway also plays a fundamental role in senescence associated with NAD^+^ deficiency, another well-established hallmark of aging. NAD + supplementation has been demonstrated to reduce cellular senescence, partly by normalizing the activation of the cGAS-STING pathway in an AD mouse model [55].

SASP plays a pivotal role in enhancing the pro-aging aspects of senescence and contributes to the accumulation of senescent cells. The composition of SASP varies across different cell types and depends on the specific senescence-inducing stimuli, but most of the factors are regulated by NF-κB pathway, at least partially via cGAS-STING activation [13, 18, 84, 85]. Importantly, a recent study by Victorelli et al. [57] directly shows that cGAS–STING activation, triggered by mtDNA release, is a potent driver of SASP. During cellular senescence, a process known as minority mitochondrial outer membrane permeabilization (miMOMP) occurs in a subset of mitochondria, leading to the release of mtDNA into the cytosol. Cytosolic mtDNA in turn activates the cGAS–STING pathway and drives SASP [57]. The cGAS-STING pathway also activates the expression of another key SASP component, IFN-I, through TBK1 autophosphorylation. De Cecco et al. showed that IFN-I can also be activated by LINE-1 elements in a cGAS-STING-dependent manner in late senescence and aging [19]. Additionally, the IFN-I signature in the choroid plexus has been shown to facilitate the age-dependent cognitive decline [86]. SASP factors propagate senescence by inducing and sustaining chronic inflammatory responses. For example, the IL-6-related cytokine, oncostatin-M, induces senescence via activating TGF-β/Smad3 through the STAT3 pathway [87]. Consistently, neutralization of IFN-β alleviates cellular senescence induced by DNA DSBs [88]. Given its central role in regulating SASP, the cGAS-STING pathway emerge as an attractive target to mitigate the SASP-induced inflammation and the associated conditions.

Aging is the biggest risk factor for neurodegenerative disorders, such as AD, FTD, and PD and evidence of senescence is found in both human patients and animal disease models. Specifically, the accumulation of senescent glial cells, including microglia, astrocyte, and oligodendrocyte progenitor cells, has been implicated in Aβ and tauopathy models of AD, resulting in inflammation and cognitive deficit [79, 89]. One of the sources of senescence in the brain is NF-κB-mediated immune response to DNA DSBs. In neurons with compromised genome integrity and heightened DSBs, the detection of elevated cGAS expression suggests its role as an upstream regulator that activates the NF-κB pathway in an AD model [90].

Gulen et al. [56] have also highlighted that cGAS-STING pathway can be activated by the cytosolic DNA released from perturbed mitochondria in aged microglia. Activation of cGAS-STING pathway alone shifted microglia to an aging and disease-related state, resulting in compromised neuronal survival and cognitive impairment. STING inhibition by H-151 reduced the levels of immune-related genes in the brains of aged mice and improved their physical and cognitive functions. The authors further created a tamoxifen-induced, microglia-specific cGAS activation mouse model and showed that tamoxifen injection resulted in cognitive impairment, increased brain inflammatory gene expression, IFN-I response, and a reduction in hippocampal neuron density. Single-nuclei RNA sequencing revealed that microglia cGAS activation also induced IFN-related transcriptomic shifts in oligodendrocytes and astrocytes. This study further supports the pivotal role of cGAS-STING, specifically in microglia, as a potent driver of aging-related neuroinflammation and neurodegeneration [56].

In the context of ataxia-telangiectasia, a neurodegenerative disorder marked by chronic inflammation, neurodegeneration, and premature aging, cGAS has a direct role in initiating the SASP linked to chronic inflammation. In a human brain organoid model of ataxia-telangiectasia, suppressing the cGAS-STING pathway resulted in a reduction of the SASP and its related neuropathology [91].

Given its strong implications in neurodegenerative disorders and cognitive decline, cellular senescence has been an attractive target for therapeutics, and cGAS presents itself as a potential target for stopping the propagation of senescence and slowing down the diseases associated with senescence.

## Therapeutic potential of cGAS-STING inhibition

cGAS and STING proteins are two key targets of the innate immune cGAS-STING signaling pathway [92, 93]. The cGAS enzyme is a structural homolog of the bacterial DncV (dinucleotide cyclase in *Vibrio*) and both enzymes belong to the cGAS/DncV-like cyclic dinucleotide synthase (CD-NTase) superfamily [94]. These enzymes possess architecture similarity to another NTase, 2′5′-oligoadenylate synthase (OAS1) [95], which is a genetic risk factor for AD and severe COVID-19 outcomes [96], and produce diverse cyclic and oligonucleotides. While cGAS synthesizes 2′,3′-cGAMP, bacterial DncV mainly produces 3′,3′-cGAMP, cyclic-di-GMP (CDG), and cyclic-di-AMP (CDA), and OAS1 produces 2′5′-oligoadenylate. All of these cyclic and oligonucleotides bind and activate the STING protein, along with various other receptors, to promote a wide range of physiological response [97–99]. STING, a trans-membrane protein, possesses a ligand-binding domain (LBD) that exists as a constitutive dimer [100, 101]. Upon binding to the cGAMP ligand, STING protein undergoes a significant conformational change, forming an oligomeric structure that ultimately facilitates the TBK1 phosphorylation of the IRF3 protein.

### cGAS inhibition

The cGAS enzyme has been suggested as an important therapeutic target in multiple human diseases, including neurodegeneration and autoimmune diseases [93]. Over the last few years, numerous cGAS inhibitors to block cGAS activity have been developed [102]. Most inhibitors were developed synthetically [103], but some natural products also inhibit cGAS [104]. Additionally, a few anti-sense oligonucleotides designed to reduce cGAS expression have been developed [105, 106]. It is worth noting that, as of now, no cGAS inhibitor has entered clinical trials to assess their effects on neurodegenerative diseases or other conditions.

Two primary subtypes of cGAS inhibitors have been developed to block cGAMP synthesis and its impact on IFN expression, as demonstrated in THP-1 myeloid cell models. [105]. One inhibitor subtype targets the cGAS active site [107–110], and the second targets the cGAS-dsDNA interaction either through binding directly to the enzyme or to dsDNA [111, 112]. Other strategies, such as blocking cGAS dimerization, post-translational modification of the N-terminal dsDNA binding domain, or an indirect regulation by other targets, have also been explored [113–115]. In this section, we briefly outline the properties of selected active site cGAS inhibitors and their potential application as experimental compounds for mechanistic studies and therapies to treat neurodegenerative diseases (Table 1). For readers interested in allosteric inhibitors and all other inhibitors, several excellent reviews describe agonist and antagonist of cGAS-STING pathway for further reading [105, 106].

**Table 1:** Representative tool cGAS inhibitors for mechanistic studies, in vitro and in vivo, and as lead compounds for developing therapies to treat neurodegenerative diseases

Organization	Compounds	Activity, properties, and comments
Rockefeller University	RU.521 [107]	Inhibits mouse cGAS with sub micromolar activity in cell-free assays and cGAS-dependent IFN induction in cellular assays. Likely brain impermeable. Suitable as experimental compounds to test in vitro
Rockefeller University/Tri-Institutional Therapeutics Discovery (TDI)	TDI-6570 [109]	Inhibits mouse cGAS with low nanomolar activity and human cGAS with a high nanomolar activity in cell-free assays, and cGAS-dependent IFN induction in mouse microglial BV2 cells at low micromolar concentration (IC_50_ = 1.64 µM) [47], but not in human macrophage THP-1 dual cells (IC_50_ > 40 µM) [116]. Brain permeable but quickly metabolized [47, 118]. Suitable as experimental compounds to test in vitro and in vivo using mouse models [47]
Rockefeller University/TDI	G-140 (TDI-8077), G-150 (TDI-8087), TDI-8246 [109], and TDI compounds [67, 68, 116, 117]	TDI-6570 analogs. Possess low nanomolar activity against h-cGAS and weakly to m-cGAS in cell-free assays and low micromolar concentrations in THP-1 dual cells. compounds. 67 and 68 show sub micromolar inhibition of h-cGAS in cell-free assays and similar activity on cGAMP production in THP-1 cells [119]. Suitable as experimental compounds to test in vitro using human iPSC-derived cells and development for therapies to treat neurodegenerative diseases
Ventus Therapeutic	Compound 102 [120]	Modified TDI-6570 analogs. Possess low nanomolar IC_50_ in cell-free assay. Shows superior activity than TDI-6570 in THP-1 dual cells. Suitable as experimental compounds to test in vitro using human iPSC-derived cells and development for therapies to treat neurodegenerative diseases
Weill Cornell Medicine	ADI034-4, ADI046-3, ADI051-5 [118]	Benzofuran and benzothiophene derivatives. Possess inhibitory effects on cGAS activity with low to high micromolar activity. ADI034-4 shows activity comparable to TDI 8246, in cell free assay [118]

Exciting progress has been made in the development of inhibitors for murine or human cGAS, considering the substantial differences between these two orthologs [121]. These distinctions include variations in as many as 116 amino acid residues within their enzymatic domains, resulting in less than 60% overall sequence similarity between the two [122]. One notable cGAS inhibitor, PF-06928215, was initially identified by the Pfizer group in 2017 using a novel fluorescence polarization assay [108]. Subsequently, more potent analogs were discovered and further refined by Aduro Biotech, resulting in analogs with novel pharmacophores [123, 124]. Additionally, Boehringer Ingelheim International Gmbh and Bellbrook lab described cGAS inhibitors with a substituted benzofuro[3,2-*d*]pyrimidine pharmacophore [125, 126], and Bellbrook lab and Immune Sensor, Inc, reported quinoline derivatives [126, 127]. These pharmacophores were also described by others [109]. Several other pharmacophores have been explored, including biphenyl carboxylic acid derivatives [128], benzimidazoles [129, 130], indazoles [131], and pyrimidone, malonitrile and sulfamoylamide derivatives [132–134].

Some of the most potent cGAS inhibitors against mouse and human cGAS were identified in two separate high-throughput screening studies with mouse and human cGAS enzymes [107, 109]. Notably, TDI-6570, one of the most potent inhibitors for mouse cGAS, possesses good brain permeability and has been used to determine the effects of cGAS inhibition on cGAS-STING pathway in a mouse model of tauopathy [47]. Meanwhile, a potent human cGAS inhibitor, G-001, and its derivatives G-140 (TDI-8077), G-150 (TDI-8087), and TDI-8246, have demonstrated more efficient inhibition of human cGAS compared to the mouse counterpart [109]. However, detailed knowledge about their pharmacokinetic properties within the human brain remains limited.

### STING inhibition

The STING protein acts as a facilitator of the innate immune signaling and is activated by cGAMP [13, 135]. The activation of STING has been shown to enhance antitumor immunity, leading to the development of various STING agonists, including small molecules, for potential cancer treatments. [136, 137]. Blocking STING activation using STING antagonists/inhibitors, on the other hand, would reduce uncontrolled immune response and could be used to treat multiple diseases, including neurodegenerative diseases [93] (Table 2). Modulation of STING activity can be achieved by targeting the LBD of the STING protein or its post-translational modifications. Indeed, the development of STING antagonists started several years ago when Aduro Biotech described cyclic-di-nucleotide (CDN) compounds, but not much was done until recently [135, 137]. The STING antagonists targeting the LBD include tetrahydroisoquinoline derivatives [138], a cyclopeptide natural product, Astin C [139], and a series of sulfonamide derivatives such as SN11, one of the most potent STING inhibitors identified so far [140]. Palmitoylation at C88/91 is a post-translational modification that activates the STING protein and is inhibited with nitrofurans and indole ureas (such as compound H-151) [141], nitro fatty acids [142], and acrylamides [143]. Other compounds that modulate cGAS-STING pathway through an unknown mechanism include butanolide heterodimers recently described by Huffman et al. [144].

**Table 2:** STING antagonists [93, 137, 145, 146]^a^

*Compounds*	*Examples, activity, and comments*
*Nitrofurans* [141]	*C-176, C-178.* Sub-µM and low nM potent. Inhibit mouse STING. Analogs active for both mouse and human STING. Act by covalently reacting through Cys-91 of STING to block Cys-91 palmitoylation
*Indole, azaindole, benzimidazole, and related compounds* [141, 147–153]	*H-151*. µM, sub-µM and low nM potent. Include indole and azaindole urea, squaramide, oxalamide, amide, and other indole derivatives. Benzimidazole derivatives described in Shen, et al. [145], as a patent. Indole H-151 blocked Cys-91 palmitoylation
*Amidine* [154]	*BB-Cl-amidine*. Inhibits STING dimerization through modification of Cys-148. Known peptidylarginine deaminase (PAD) inhibitor
*Tetrahydro-iso-quinolones* [138]	An optimized tetrahydro-iso-quinoline carboxylic acid inhibits STING with 68 nM. Shows EC50 > 10 µM in cGAMP-induced IFNβ production in THP1 cells
*Cyclic peptide* [139]	*Astin C,* low µM potent. Binds to the C-terminal domain of STING and possibly the CDN binding pocket
*Amido-benzene-sulfonamides* [140]	SN-011. Sub-µM potent. Identified and developed by virtual HTS and modification of a hit amido-benzene-sulfonamide compound. Binds and inhibits through the CDN binding pocket
*Alkaloid* natural product [155]	*Gelsevirine.* High sub-µM potent. Targets CDN binding domain and inhibits cGAMP activity. Promotes K48-linked STING ubiquitination and degradation
*Others* [145, 146]^a^	Include dimeric benzimidazoles, dimeric furan-2-ones, 6,5-heterocyclic derivatives, purines, diadenosine triphosphate, and STING degraders

^a^See additional references in the patent review by Shen, et al., Expert Opin Ther Pat, 2022. 32(11): p. 1131–1143

At the time this review was written, no cGAS or STING inhibitors were in clinical use. Given the exciting progress in the development of cGAS inhibitors and safety profile of genetic models with cGAS loss of function, we believe that cGAS inhibitors hold great therapeutic potential for various neurological disorders.

## Conclusions and outlook

The cGAS-STING pathway has emerged as a critical player in the CNS, contributing to various neurological disorders characterized by chronic neuroinflammation. Targeting this offers the potential to modulate immune responses, alleviate inflammation, and restore immune homeostasis in various pathological conditions. As our understanding of the role of the cGAS-STING pathway in CNS continues to expand, several exciting avenues for future research and clinical applications emerge.

Firstly, further investigation is needed to elucidate the precise molecular mechanisms underlying cGAS-STING activation and downstream signaling in CNS cell types other than microglia (e.g., neurons, endothelial cells, and peripheral immune cells). Unraveling these intricacies will enhance our understanding of the diverse functions of the cGAS-STING pathway in CNS homeostasis and pathology.

Moreover, exploring the crosstalk between the cGAS-STING pathway and other signaling pathways implicated in CNS diseases can provide valuable insights into disease pathogenesis and potential therapeutic targets. For instance, studies have revealed the crosstalk between cGAS-STING and autophagy and lipid metabolism pathways, both of which are highly dysregulated in neurodegenerative diseases [156, 157].

Additionally, the development of selective and potent modulators targeting the cGAS-STING pathway holds promise for therapeutic interventions. cGAS and STING are presumably the most obvious and straightforward targets that are inhibited using small molecules. Selective activation or inhibition of other targets/pathways or processes, including TREX1 catalyzed degradation of dsDNA [158], ENPP1 (Ecto-nucleotide pyrophosphatase phosphodiesterase 1) mediated degradation of cGAMP [159], and TBK1-STING-IRF3 interactions [12], could be carefully examined as the potential therapeutic strategies. Small-molecule inhibitors or activators that specifically target such key components of the cGAS-STING pathway could offer potential treatments for CNS disorders characterized by chronic neuroinflammation. However, careful consideration of the potential side effects and off-target effects of such interventions is necessary.

In conclusion, the role of the cGAS-STING pathway in the CNS represents an exciting area of research with significant therapeutic implications. Further exploration of its molecular mechanisms, crosstalk with other pathways, and development of targeted interventions could provide novel treatments for a broad range of CNS diseases characterized by chronic neuroinflammation. The future of cGAS-STING research in the CNS holds great promise for advancing our understanding and improving patient outcomes in the field of neurological disorders.

## Data Availability

NA.

## Abbreviations

Aβ: β-Amyloid
AD: Alzheimer’s disease
ALS: Amyotrophic lateral sclerosis
APC: Antigen-presenting cells
BAK: BCL-2 homologous antagonist/killer
BAX: BCL2 associated X protein
CCF: Cytoplasmic chromatin fragments
CCI: Controlled Cortical Impact
CCR5: C–C chemokine receptor type 5
CDA: Cyclic-di-AMP
CDG: 3′,3′-CGAMP, cyclic-di-GMP
CDN: Cyclic-di-nucleotide
CD-NTase: Cyclic dinucleotide synthase
CNS: Central nervous system
cGAMP: Cyclic GMP-AMP
cGAS: Cyclic GMP-AMP synthase
DAMP: Damage-associated molecular pattern
dsDNA: Double-stranded DNA
DncV: Dinucleotide cyclase in *Vibrio*
ENPP1: Ecto-nucleotide pyrophosphatase phosphodiesterase 1
FTD: Frontotemporal dementia
HD: Huntington’s disease
HSV-1: Herpes simplex virus
HTT: Huntingtin gene
IFN-I: Type I interferon
IFNAR: IFN-I receptor
iPSCs: Induced pluripotent stem cells
IRF3: Interferon regulatory factor 3
ISG: Interferon-stimulated gene
JAK: Janus kinase
LBD: Ligand binding domain
LINE-1: Long-interspersed element-1
MEF2C: Myocyte enhancer factor 2C
mHTT: Mutant HTT
miMOMP: Minority mitochondrial outer membrane permeabilization
mPTP: Mitochondria transition pore
MPTP: 1-Methyl-4-phenyl-1, 2, 3, 6-tetrahydropyridine
mtDNA: Mitochondrial DNA
NLRX1: NLR containing X1
OAS1: NTase, 2′5′-oligoadenylate synthase
PD: Parkinson’s disease
PQBP1: Polyglutamine binding protein 1
PRRs: Pattern recognition receptors
SA-β-Gal: Senescence-associated β-galactosidase
SASP: Senescence-associated-secretory-phenotype
SCAPs: Senescent cell anti-apoptotic pathways
SETX: RNA–DNA helicase senataxin
SOD1: Superoxide dismutase 1
STAT: Signal transducer and activator of transcription
STING: Stimulator of interferon genes
αSyn-PFF: αSyn preformed fibril
TBI: Traumatic brain injury
TBK1: TANK-binding kinase 1
TFAM: Transcription factor A, mitochondrial
TH: Tyrosine hydroxylase
VDAC: Voltage-dependent anion channels
